# A Military Veteran with Pulmonary Fibrosis, Hepatic Cirrhosis, Bone Marrow Dysfunction and Shortened Telomeres Found to Have GAVE, a Potential Unsuspected Consequence of Telomere Spectrum Disorder

**DOI:** 10.3390/jcm15187073

**Published:** 2026-09-11

**Authors:** Anton Uhlen, Omar A. Oudit, Darshan Patel, Niraj Shah, Lena Delorenzo, Shreya Vuchula

**Affiliations:** 1 Jefferson Health New Jersey, Stratford, NJ 08084, USA; antonuhlen11@gmail.com (A.U.); darshan3165@gmail.com (D.P.); niraj.shah@jefferson.edu (N.S.); lena.delorenzo@jefferson.edu (L.D.); shreya.vuchula@jefferson.edu (S.V.); 2 One Brooklyn Health Brookdale University Medical Center, Brooklyn, NY 11212, USA

**Keywords:** telomere spectrum disorder, acquired shorted telomere dysfunction, pulmonary fibrosis, cryptogenic cirrhosis, gastric antral vascular ectasia, cytopenias, Gulf War Syndrome

## Abstract

**Background:** Telomeres are repetitive DNA sequences at chromosome ends that maintain genomic stability and shorten with age. Mutations in genes such as DKC1, TERC, TERT, NOP10, TINF2 and NHP2 cause Telomere Spectrum Disorders (TSD), leading to shortened telomeres. Acquired TSDs arise from environmental or occupational exposures and commonly affect veterans, truck drivers, and industrial workers. These exposures generate reactive oxygen species associated with cancer, liver disease, pulmonary disease, and bone marrow complications. Idiopathic Pulmonary Fibrosis (IPF) is most common pulmonary manifestation, followed by hepatic fibrosis/cirrhosis, hematologic disorders (e.g., aplastic anemia, MDS), and rarely gastrointestinal findings. This case describes a Gulf War veteran with suspected acquired short telomeres and multisystem involvement (idiopathic cirrhosis, pulmonary fibrosis, cytopenias) and uniquely found to have gastrointestinal (Gastric Antral Vascular Ectasia, GAVE), a potential unsuspected manifestation of TSD. **Methods/Case:** A 76-year-old male with atrial fibrillation (post-Watchman), cryptogenic cirrhosis, COPD, IPF, diabetes, and anemia presented with severe shortness of breath and hypoxia. Admitted for Sepsis and Acute Hypoxic Respiratory Failure. Treated with AVAPS, IV Zosyn, IV Lasix, steroids, bronchodilators, and diuretics. His condition improved, and he was discharged on baseline 3L O2. His Gl history included GAVE, an unsuspected bleeding complication causing chronic iron-deficiency anemia. Hematologic findings included chronic anemia, thrombocytopenia, and a hypocellular bone marrow with 12% atypical NK cells. **Discussion/Results:** The patient’s Gulf War exposure to oil fire pollutants (benzene, toluene, PAHs, lead, cadmium, and particulate matter) likely contributed to telomere shortening and TSD-related multisystem disease. Given his shortened telomeres, testing for TERC and TERT mutations is warranted. GAVE (“watermelon stomach”) is a rare cause of GI bleeding characterized by dilated gastric vessels and chronic anemia and has been linked to TSD but no current reports have documented this association. Its occurrence in this patient suggests an unrecognized gastrointestinal manifestation of TSD as the patient does not have known history of portal hypertension. **Conclusions:** This represents an potentially underreported manifestation of acquired TSD presenting with combined hepatic, pulmonary, hematologic, and gastrointestinal (GAVE) involvement.

## 1. Introduction

**Figure 1:**
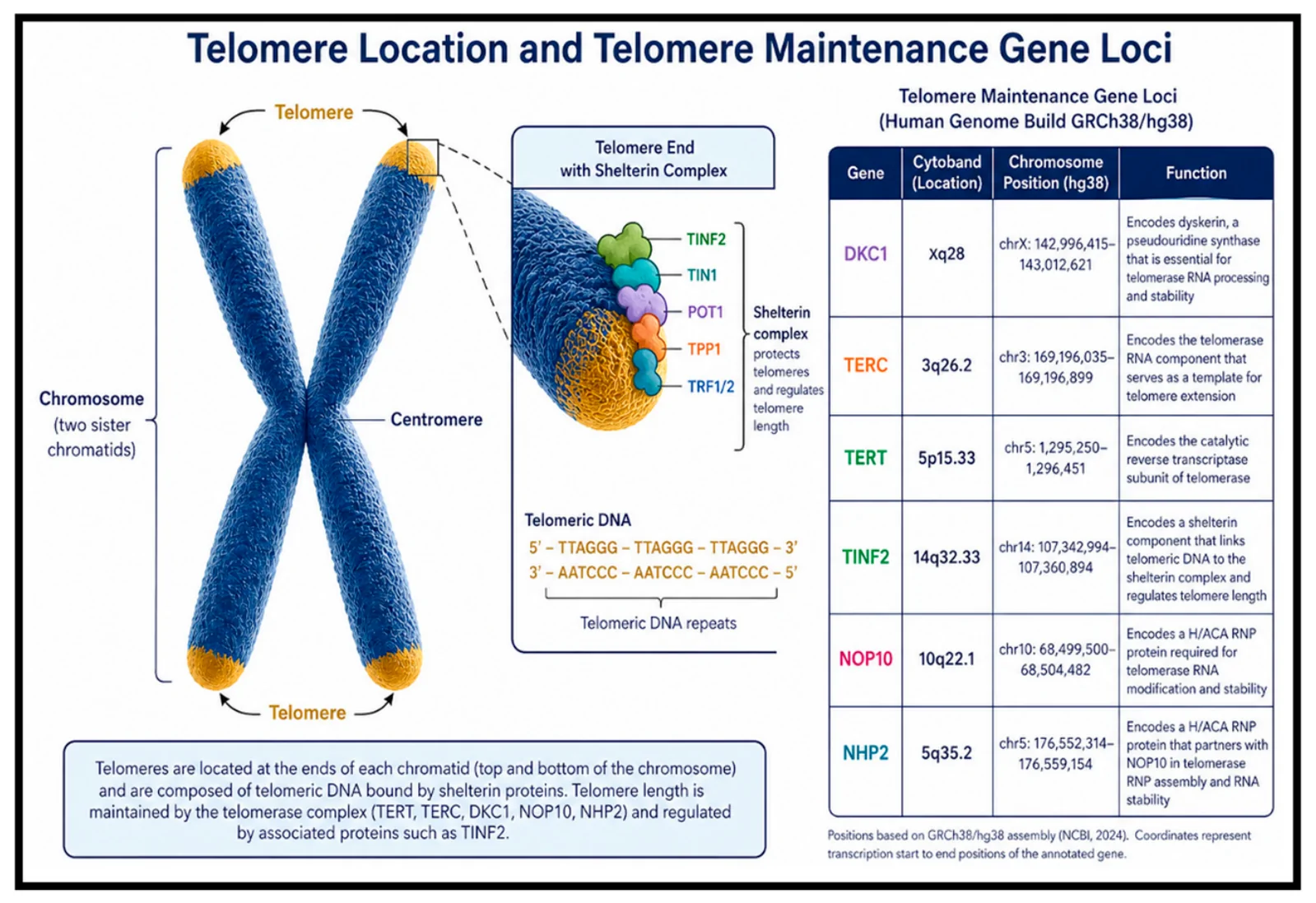
Telomere Location and Telomere Maintenance Gene Loci. Figure 1 illustrates and defines a telomere and the predicted loci of the DKC1, TERC, TERT, TINF2, NOP10, and NHP2 alleles.

Telomeres are terminal DNA sequences located at the ends of chromosomes that identify biological age. They are repetitive six-nucleotide sequences that protect chromosome integrity during the cell cycle, and they also maintain genomic stability [1,2]. While telomeres shorten with age due to repeated cellular mitosis, the clinical implications of shortened telomeres are known as a spectrum of disorders called shortened telomere disorders or telomere biology disorders [1,2]. The literature has identified six specific genetic mutations (DKC1, TERC, TERT, TINF2, NOP10, and NHP2) responsible for 50–60% of all inherited disorders, most significantly dyskeratosis congenita [1,2,3,4,5,6]. Figure 1 shows the common telomere genes and their location within chromosomes [1,2,3,4,5,6].

Alternatively, acquired short telomere disorders are associated with a variety of environmental and occupational risk factors. Major risk factors include exposure to particulate matter, black carbon, benzene, toluene, polycyclic aromatic hydrocarbons, N-nitrosamines, pesticides, lead, and other hazardous waste [7]. Occupations such as truck drivers, industry workers, mechanics, military veterans, and many others are at high risk of exposure [7]. These environmental and occupational chemical air pollutants have been associated with cardiac and pulmonary morbidity and mortality, given the increased formation of reactive oxygen species, which have been linked to shortened telomeres [7].

**Figure 2:**
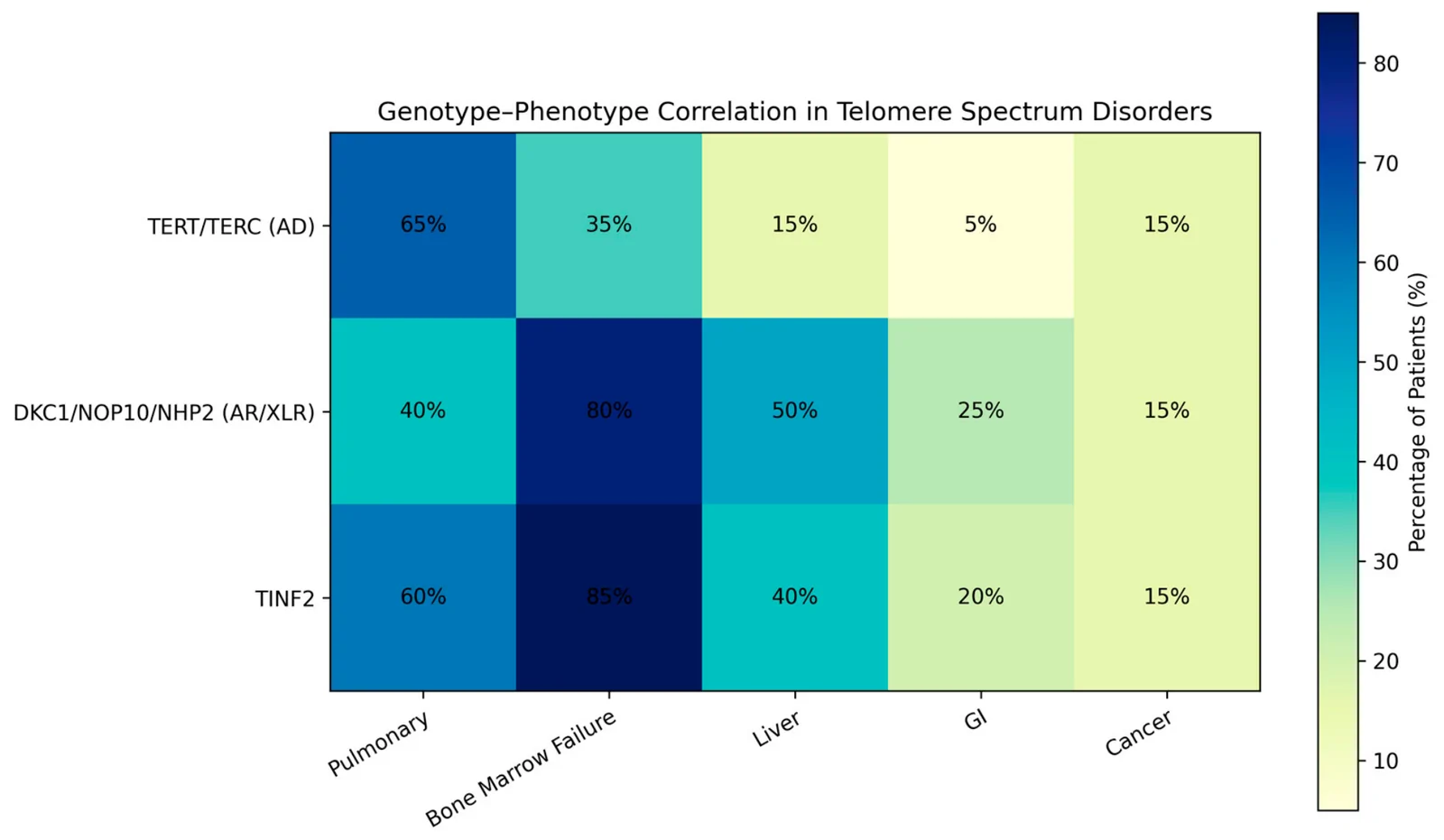
Heat map genotype–phenotype correlations of patients with TSD based on common genetic mutations. The heat map summarizes approximate phenotype prevalence across major telomere-associated genotype groups. TERT/TERC gene mutations are associated with autosomal dominant disease and demonstrate a predominantly pulmonary phenotype, with pulmonary fibrosis representing a major adult manifestation [2,4]. In contrast, DKC1/NOP10/NHP2-associated AR/XLR disease is characterized by greater multisystem involvement, including bone marrow failure, liver disease, and gastrointestinal manifestations [1,2,5]. TINF2-associated disease is associated with severe early-onset hematopoietic and pulmonary involvement [1,5].

Acquired telomere genetic mutations accumulate with respect to factors such as increased age, male sex, stress, obesity, alcohol consumption, environmental exposure, and smoking, which have been shown in some studies to cause advanced telomere degradation. The association between these needs further evaluation, as the correlation remains weak [7,8]. However, while advanced age results in shorter telomeres, mutations within these genes responsible for maintaining telomeres, such as TERC and TERT, have been shown to be frequent in cancers, increasing the risk for malignancies and thus resulting in a wide array of complications [9]. The literature has linked telomere gene mutations (both inherited and acquired) or shortened overall telomere length to an array of malignancies [10,11], liver disease [10,12,13], pulmonary disease [10,12,13,14], and hematologic manifestations or bone marrow failure [10,12]. Figure 2 illustrates a heat map showing the frequency of developing these manifestations based on their associated telomere mutation.

Hepatic, pulmonic, hematologic, and GI manifestations have been shown in the literature to be associated with telomere disorders, with hepatic and pulmonic manifestations being the most frequent [10,12,14]. When pulmonic manifestations of shortened telomere syndrome with moderate mutations are present, typically TERC/TERT mutations, they typically present first and present in up to 65% of patients with telomere disorders [12,14]. These pulmonic manifestations are idiopathic pulmonary fibrosis [1,2,10,12,13,14] (progressive and irreversible pulmonary scarring that results in inevitable respiratory failure) or smoking-related pulmonary emphysema with or without fibrosis [10,14]. PFT and CT imaging are needed for diagnosis, and imaging findings consistent with pulmonary fibrosis are patchy, basal, and peripheral reticular opacity, ground-glass opacity, and honeycombing [12]. Meanwhile, hepatic manifestations of telomere disorders are frequently cirrhosis, cryptogenic liver fibrosis, or portal hypertension [1,2,10,12,14], and they are also associated with TERC or TERT mutations [10,15,16]. Typical initial presentation is with asymptomatic transaminitis, nonspecific ultrasound findings, or abnormal biopsy results, including heterogeneous inflammation, iron accumulation in the absence of transfusional or hereditary hemochromatosis, hepatocyte necrosis, bridging f ibrosis, or nodular regenerative hyperplasia [10,12]. Multiple reports have also linked hepatopulmonary syndromes to telomere disorders [1,2,10,13], such as alpha 1 antitrypsin deficiency (a cause of pulmonary emphysema) [14]. However, in most reports, telomere disorders with both GI and pulmonary manifestations tend to be inherited [1,2,13] rather than acquired. Finally, hematologic manifestations, such as aplastic anemia or myelodysplastic syndromes, as well as bone marrow suppression, can be present in telomere disorders [2,10,14], but the etiology of this is unclear, as hepatic and pulmonary manifestations can also contribute to some of the hematologic manifestations. Additionally, gastroenterological manifestations such as esophageal and lacrimal duct stenosis, enteropathy, and enterocolitis have been described in some studies, but these lack convincing evidence [12].

Here, we present a unique case of an acquired short telomere disorder in a military veteran exposed to toxic gases during the Gulf War who presented with coagulopathies, idiopathic pulmonary fibrosis, hepatic cirrhosis, and GAVE (a rare GI side effect) with confirmed acquired short telomere syndrome. To our knowledge, this is the first case in which a patient with acquired short telomere disorder presented with all major findings of known telomere disorders, resulting in hepatic, pulmonary, hematologic, and GI (GAVE) symptoms together.

## 2. Case Presentation

**Figure 3:**
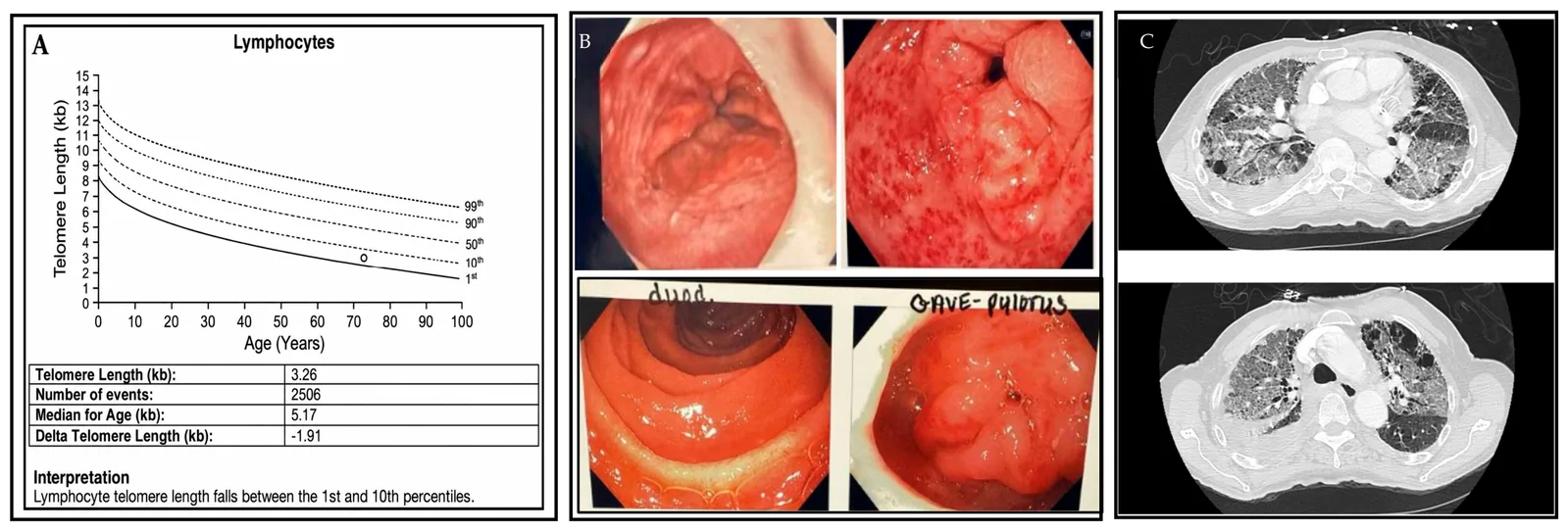
Genetic Evaluation confirming shortened telomeres, endoscopic evaluation confirming GAVE and CT imaging findings confirming COPD and extensive fibrosis. (**A**) shows the confirmed shortened telomeres seen on genetic evaluation. (**B**) shows the endoscopic confirmation of GAVE. (**C**) shows two cross sections evaluated on CT imaging during the current hospital visit showing multifocal pneumonia, pulmonary vascular congestion, underlying chronic COPD changes, and fibrotic lung disease.

This case focuses on a 76-year-old male military veteran (formerly part of Desert Storm during the Gulf War) with a medical history significant for atrial fibrillation status post Watchman procedure, cryptogenic cirrhosis, chronic obstructive pulmonary disease (COPD), idiopathic pulmonary fibrosis (IPF) on 3L NC at home, noninsulin-dependent diabetes mellitus, acquired TSD (Figure 3A), GAVE diagnosed on EGD (Figure 3B), and chronic anemia with confirmed bone marrow suppression presented with worsening shortness of breath and hypoxia. Initial imaging demonstrated findings concerning for multifocal pneumonia, pulmonary vascular congestion, underlying chronic COPD changes, and fibrotic lung disease (Figure 3C). Laboratory evaluation combined with radiologic evaluation demonstrated severe sepsis secondary to pneumonia. The patient was admitted and empirically started on antibiotics, given systemic corticosteroids with bronchodilators and placed on AVAPS ventilation for his acute hypoxic respiratory failure. Pulmonology and cardiology were consulted and followed the patient throughout the hospital stay. The patient’s hospital course was complicated by multiple episodes of fluctuating respiratory status with increased oxygen requirements but did not require intubation. His condition gradually improved over a few days, and he was ultimately stable for discharge on his baseline oxygen requirement of 3 L via nasal cannula.

Adding clinical context to this case, this patient had developed cryptogenic cirrhosis without any known clinical risk factors or evidence of alcohol abuse, obesity, hepatitis, or known cirrhotic markers. CT imaging of the abdomen and pelvis confirmed upper abdominal varices and cirrhosis. He was a former smoker (10 PPY) but developed COPD and fulminant pulmonary fibrosis (Figure 3C). This patient was previously worked up for iron deficiency anemia, requiring endoscopic evaluation treated with endoscopic banding x5 one week prior to presentation; no old blood or active bleeding was noted, and he had also previously been treated with argon plasma coagulation during EGD before that. Images confirming GAVE on endoscopy are shown in Figure 3B. This patient did have multiple prior EGDs and was found to have GAVE without any form of portal hypertension or abdominal varices noted. Before this hospital stay, the patient had undergone telomere length analysis via flow cytometry and FISH. His shortened telomeres were confirmed to be <10% (Figure 3A). Flow cytometry and FISH analysis are the first-line exams for evaluating TSD [17]. For this patient, flow cytometry and FISH were performed on peripheral leukocytes, which is the standard first-line approach [17]. Telomere length reference values were all based on the laboratory standard (Johns Hopkins Genomics) and compared to that of the previous published literature and guidelines [18,19]. Finally, the patient had a history of thrombocytopenia and anemia, evaluated by hematology multiple times. The patient underwent bone marrow evaluation in 2025. Flow cytometric analysis detected hypocellular bone marrow relative to age and 12% atypical NK cells. All three major cell lines remained intact despite persistent anemia and thrombocytopenia previously requiring multiple transfusions.

## 3. Discussion

It is well documented that during the Gulf War, there were many oil-well fires, exposing soldiers to a variety of environmentally hazardous pollutants including benzene, toluene, polyaromatic hydrocarbons, heavy metals (most commonly lead and cadmium), and fine particulate matter [17]. These exposures have been linked to telomere-associated disorders and many cardiac and pulmonary manifestations [7]. Some refer to this collection of exposures as Gulf War Syndrome, but this is a diagnosis of exclusion and identifying causality is difficult. Given that this patient is a veteran who served in the Gulf War and had been exposed to these chemicals, his risk for pulmonary morbidity and mortality secondary to hazardous air pollutants is significantly increased, as well as his risk for telomere-associated disorders. A <10th percentile for age and especially <1st percentile for age supports a diagnosis of a shortened telomere syndrome. [20] This is also corroborated by the literature illustrating approximately ~30 base pairs shortening annually as we age, which is in line with the 50th percentile as illustrated in Figure 3A [21]. Indeed, this patient also has risk factors and confirmed shortened telomeres (given that his telomeres fell in the 1st–10th percentile based on age) based on genetic evaluation, adding to the acquired diagnosis. He also had confirmed multisystem involvement, including pulmonary fibrosis, cryptogenic cirrhosis, and bone marrow suppression, which are known clinical manifestations of TSD. Finally, this patient’s mid-60s age of onset argues against a germline telomere spectrum disorder, since a study of 231 participants found median onset ages of 36.1 years for autosomal, 11.3 years for autosomal recessive and X-linked, and 8.1 years for TINF2-associated TSD [3]. Combined with his Gulf War environmental exposure to known carcinogens, his demographic and clinical presentation favors an acquired, rather than germline, etiology.

Given that the previous literature has linked telomere shortening to impaired endothelial repair and increased vascular fragility, this may contribute to the development of ectatic vascular lesions such as GAVE and represent an underrecognized manifestation of TSD that, to our knowledge, has not been previously explored [8,18,19]. Abdominal varices and portal hypertensive changes are well-established manifestations of cirrhosis and portal hypertension [22,23]. In our patient, these findings of abdominal varices and portal hypertension were therefore attributed to progression of his underlying cirrhosis. Notably, during an EGD performed in three years before this presentation, the findings demonstrated GAVE without evidence of esophageal varices or portal hypertensive changes, indicating that GAVE preceded the subsequent development of clinically apparent portal hypertension. Although GAVE is frequently observed in patients with cirrhosis, it is pathophysiologically distinct from portal hypertensive gastropathy and does not necessarily reflect the presence or severity of portal hypertension [22,23]. In the context of this patient’s telomere biology disorder, the earlier development of GAVE may warrant additional consideration, as gastrointestinal hemorrhage, telangiectasias and vascular abnormalities have been described in telomere spectrum disorder. One small study with pediatric children and known TSD pathogenic TINF2 variant mutations illustrated these findings [24]. Therefore, this raises the possibility that vascular ectasia abnormalities may represent an additional phenotypic manifestation of telomere dysfunction, but this has not been shown in adults or in acquired mutations.

Given the extensive literature indicating that genetic mutations such as TERC and TERT could result in telomere fragility, a genetic evaluation for any of the mutations previously discussed would be the next step in confirming the diagnosis of adult-onset acquired TSD. Finally, the patient had no known history of portal hypertension noted on EGD, which addresses the confounding bias of cirrhosis and portal hypertension in the development of GAVE.

## 4. Conclusions

Overall, this case illustrates an example of an acquired TSD with multisystem involvement including hepatic, pulmonary, and hematologic manifestations, which could serve as an argument for unexplained vascular manifestations (i.e., GAVE) not previously evaluated. Limitations of this case include the single-patient design, absence of confirmatory germline mutation, genetic testing confirming one of the previous telomere genetic mutations (i.e., TERT or TERC), and the inability to establish causality between environmental exposure, telomere shortening, and GAVE. Confirmatory testing for targeted gene mutation would need to be completed using germline DNA sequencing, which was not completed for this patient [21]. That being said, any combination of pulmonary fibrosis, cryptogenic cirrhosis, cytopenias, GI manifestations, or unexplained vascular manifestations (i.e., GAVE) should prompt consideration for TSD.

## Data Availability

The original contributions presented in this study are included in the article. Further inquires can be directed to the corresponding author.
